
JOURNAL INFORMATION
==============================
NLM Title Abbreviation: Precis Clin Med
Journal ID: Precis Clin Med
NLM Journal ID: 101739592
Journal ID: pcm

Precision Clinical Medicine

ISSN: 2096-5303
EISSN: 2516-1571

ARTICLE INFORMATION
==============================
PMCID: PMC7499720
PMID: 35960674
DOI: 10.1093/pcmedi/pbaa031
Article ID: pbaa031
Article version: 1
Subjects: Corres, AcademicSubjects/MED00010

SARS-CoV-2 reinfection in two patients who have recovered from COVID-19

Zhang Kang kang.zhang@gmail.com Faculty of Medicine and University Hospital, Macau University of Science and Technology, Macau SAR, China

Lau Johnson Yiu-Nam Department of Applied Biology and Chemical Technology, Hong Kong Polytechnic University, Hong Kong SAR, China

Yang Lei The First Affiliated Hospital, Sun Yat-sen University, Guangzhou 510080, China

Ma Zhen-Guo Renmin Hospital of Wuhan University, Wuhan 430060, China

Correspondence: Kang Zhang, kang.zhang@gmail.com
Electronic publication date: 2020 Sep 4
Electronic Location ID: pbaa031
Copyright: © The Author(s) 2020. Published by Oxford University Press on behalf of the West China School of Medicine & West China Hospital of Sichuan University.
Copyright year: 2020
License: This is an Open Access article distributed under the terms of the Creative Commons Attribution License (http://creativecommons.org/licenses/by/4.0/), which permits unrestricted reuse, distribution, and reproduction in any medium, provided the original work is properly cited.
License URL: https://creativecommons.org/licenses/by/4.0/

Keywords: asymptomatic carriers, re-infection, COVID-19
edited-state: accepted-manuscript
article-lifecycle: PAP

==============================
**SARS-CoV-2 reinfection in two patients who have recovered from COVID-19**

Kang Zhang[1], Johnson Yiu-Nam Lau[2], Lei Yang[3], MD, Zhen-Guo Ma[4]

[1]Faculty of Medicine and University Hospital
Macau University of Science and Technology
Macau SAR, China

[2]Department of Applied Biology and Chemical Technology
Hong Kong Polytechnic University
Hong Kong SAR, China

[3]The First Affiliated Hospital
Sun Yat-sen University
Guangzhou 510080, China

[4]Renmin Hospital of Wuhan University
Wuhan 430060, China

Corresponding author: Kang Zhang, kang.zhang@gmail.com

Key words: asymptomatic carriers, re-infection, COVID-19

The duration of immune protection against SAR-CoV-2 reinfection in patients who have recovered from COVID-19 is a critical unanswered question. To determine the long term clinical and virologic outcome in patients who recovered from COVID-19, we have prospectively followed 220 COVID-19 patients admitted to Renmin Hospital of Wuhan University between January 15 and February 15, 2020 and recovered and had follow-up till July 15, 2020. Throat swab SARS-CoV-2 RNA was tested by a reverse transcription polymerase chain reaction (RT-PCR) regularly during hospitalization and follow-up[1].

Of the 220 patients, 193 patients recovered from COVID-19 infection and remained throat swab viral RNA negative during follow-up. For the 27 patients who were positive for throat swab viral RNA during follow-up, we have identified three different virologic patterns.

First, 16 patients (M:F 5:11, age 24-77) showed a positive throat swab viral RNA (usually with a weak signal, + out of +++ signal) during the first admission and discharged home when viral RNA was negative. After an average of 3.7 days (range 1-16 days), the follow-up viral RNA was positive again [usually also with a weak signal (+)] for a period of 5 to 57 days before turning negative again. As all viral RNA positive patients were required to stay in the hospital, the first admission was for 4 to 56 days and the second admission was for 5 to 57 days. COVID-19 disease severity was assessed based on the classification recommended by the Chinese Health Authority [7$^{th}$ edition; mild – asymptomatic/relatively asymptomatic with no

ORIGINAL UNEDITED MANUSCRIPT

© The Author(s) 2020. Published by Oxford University Press on behalf of the West China School of Medicine & West China Hospital of Sichuan University. This is an Open Access article distributed under the terms of the Creative Commons Attribution License (http://creativecommons.org/licenses/by/4.0/), which permits unrestricted reuse, distribution, and reproduction in any medium, provided the original work is properly cited.

CXR/chest CT changes; moderate – fever and/or respiratory symptoms or/and with radiological evidence of pneumonia (can be relatively asymptomatic but with radiologic changes); severe – severe respiratory symptoms with oxygen saturation of <93%, drop in arterial oxygen tension or CXR/Chest CT showing 50% of more of the lung areas involved; and ICU admission – respiratory distress requiring mechanical ventilation or organ failures requiring ICU care]. For the first submission, the disease severity was classified as mild 1, moderate 11, severe 4, ICU 0; and for the second submission, the severity was classified as mild 3, moderate 13, and severe and ICU admission 0. All 16 patients were symptomatic on their first submission and 9 of 16 were relatively asymptomatic during their second submission. These patients might represent intermittent viral RNA positivity during their recovery. However, the observation that the second admission was up to 57 days of viral RNA positivity and 9/16 being relatively asymptomatic highlighted the importance of virologic follow-up of the recovered patients to identify those that could turn viral RNA positive again so as to reduce the pool of infection sources.

Second, 9 patients (M:F 2:7, age 22-78) showed strong signal for throat swab viral RNA based on RT-PCR (majority +++), which turned negative for 2 to 13 days, and then turned strongly positive (+++) again for 7 to 28 days. Their disease severity was mild 0, moderate 7, severe 2, ICU 0 for their first admission (5-35 days); and severity of mild 3, moderate 6, severe 0, and ICU 0 for their second admission (for 7 to 28 days). During the first admission, 1/9 patient was relatively asymptomatic and during the second admission, 6/9 were relatively asymptomatic. Again, these subjects could serve as potential source of infection. Whether these big fluctuations of viral RNA in their throat swab represented viral RNA titer variations or whether these 9 subjects represented viral reactivation or with new viral infections were difficult to determine clinically.

Third, in two patients (both F and both 33 years old), their first admission showed disease severity of 1 moderate and 1 severe and had hospital stay of 16 and 38 days. Both recovered clinically and were discharged with negative throat swab viral RNA. They were clinically well and negative for viral RNA during follow-up for 43 and 58 days. Then, they became positive for throat viral RNA again, and both had a return to moderate disease severity during the second admission (66 and 75 days). Before the second admission, both patients also showed reduction in IgG anti-SARS-CoV-2 antibody[1] with one being seronegative and one weak positive before throat swab viral RNA was positive again. During the second admission, both patients showed an increase in their IgG anti-SARS-CoV2 titer and one patient also showed a renewed reactivity for IgM anti-SARS-CoV-2. As both had recovered clinically and had negative throat swab viral RNA for 43-58 days before they became sick again with positive throat swab viral RNA together with an increase in IgG antibody (with one also showing IgM again), they were considered as having SARS-CoV-2 reinfection. Whether the low/absent IgG before the reinfection predisposed these two patients to reinfection requires further clarification in a bigger cohort of follow-up subjects. However, the observation of these reinfections suggested that the concept of herb immunity protection may not be applicable in at least a small proportion of COVID-19 recovered patients.

ORIGINAL UNEDITED MANUSCRIPT

These observations highlighted first, the importance to follow-up the COVID-19 recovered patients to ensure that we can identify the small percentage of patients who may turn viral RNA positive again. As some of the patients who turned viral PCR positive again were relatively asymptomatic, they can still serve as sources for new infections[2]. Second, both clinical and virologic profiles indicated that two patients in our cohort had SARS-CoV-2 reinfection, indicating that the herb immunity approach may not protect all the recovered subjects.

Reference:

1. Xu X, Sun J, Nie S, Li H, Kong Y, Liang M, Hou J, Huang X, Li D, Ma T, Peng J, Gao S, Shao Y, Zhu H, Lau JYN, Wang G, Xu C, Jiang L, Huang A, Yang Z, Zhang K, Hou FF. Seroprevalence of immunoglobulin M and G antibody against SARS-CoV-2 in China. Nat Med (2020). https://doi.org/10.1038/s41591-020-0949-6
2. Wang T, Wu Y, Lau JYN, Yu Y, Liu L, Li J, Zhang K, Tong W, Jiang B. A four-compartment model for the COVID-19 infection—implications on infection kinetics, control measures, and lockdown exit strategies, *Precision Clinical Medicine*, Volume 3, Issue 2, June 2020, Pages 104–112, https://doi.org/10.1093/pcmedi/pbaa018

ORIGINAL UNEDITED MANUSCRIPT
